# The Clinicopathological Significance of miR-133a in Colorectal Cancer

**DOI:** 10.1155/2014/919283

**Published:** 2014-07-01

**Authors:** Timothy Ming-Hun Wan, Colin Siu-Chi Lam, Lui Ng, Ariel Ka-Man Chow, Sunny Kit-Man Wong, Hung-Sing Li, Johnny Hon-Wai Man, Oswens Siu-Hung Lo, Dominic Foo, Alvin Cheung, Thomas Yau, Jensen Tung-Chung Poon, Ronnie Tung-Ping Poon, Wai-Lun Law, Roberta Wen-Chi Pang

**Affiliations:** Department of Surgery, LKS Faculty of Medicine, The University of Hong Kong, Queen Mary Hospital, 102 Pok Fu Lam Road, Pok Fu Lam, Hong Kong

## Abstract

This study determined the expression of microRNA-133a (MiR-133a) in colorectal cancer (CRC) and adjacent normal mucosa samples and evaluated its clinicopathological role in CRC. The expression of miR-133a in 125 pairs of tissue samples was analyzed by quantitative real-time polymerase chain reaction (qRT-PCR) and correlated with patient's clinicopathological data by statistical analysis. Endogenous expression levels of several potential target genes were determined by qRT-PCR and correlated using Pearson's method. MiR-133a was downregulated in 83.2% of tumors compared to normal mucosal tissue. Higher miR-133a expression in tumor tissues was associated with development of distant metastasis, advanced Dukes and TNM staging, and poor survival. The unfavorable prognosis of higher miR-133a expression was accompanied by dysregulation of potential miR-133a target genes, LIM and SH3 domain protein 1 (LASP1), Caveolin-1 (CAV1), and Fascin-1 (FSCN1). LASP1 was found to possess a negative correlation (*γ* = −0.23), whereas CAV1 exhibited a significant positive correlation (*γ* = 0.27), and a stronger correlation was found in patients who developed distant metastases (*γ* = 0.42). In addition, a negative correlation of FSCN1 was only found in nonmetastatic patients. In conclusion, miR-133a was downregulated in CRC tissues, but its higher expression correlated with adverse clinical characteristics and poor prognosis.

## 1. Introduction

Alone, colorectal cancer (CRC) is responsible for 600,000 mortalities annually and approximately 1.2 million new cases are reported each year [1]. This makes CRC the 3rd most common cancer worldwide and the 2nd leading cause of cancer-related deaths in Europe and USA [2]. The sporadic occurrence of CRC is a combinative effect of environmental and genetic factors leading to the multistage progression from normal to adenoma and then finally to malignant carcinoma over several years to decades [3]. In approximately 20% of cases, there is a strong genetic component involved as first-degree relatives are diagnosed with the same cancer or they have an underlying genetic predisposition (e.g., diabetes mellitus, obesity, gender, and inflammatory bowel disease) that increases the risk of CRC. The main important extrinsic factor is lifestyle. In particular, diet with high intake of alcohol, red-meat and fat, and insufficient fiber uptake increases the risk for CRC [4].

MiRNAs are highly conserved, small, noncoding RNA approximately 19–25 nucleotides in length, with each capable of regulating hundreds of genes and acting as key regulators potentially affecting the expression of oncogenes and tumor suppressors [5]. They function by targeting multiple transcripts to epigenetically modulate the gene translation rate and induce messenger RNA (mRNA) degradation depending on the strength of binding to 3′ untranslated region (3′UTR). Their importance is highlighted by the fact that they are involved in almost all essential biological cellular processes including apoptosis, differentiation, proliferation, and migration, and they are suggested to regulate more than 50% of all protein-coding genes [6]. Within the Wellcome Trust Sanger Institute, release 20, in June 2013, 1872 sequences of human miRNAs have been identified thus far [7].

The potential for miRNAs as epigenetic biomarkers has long been recognized as a noninvasive method for early CRC detection to enable more effective therapeutic intervention and evaluation of survival [8]. However, the exact mechanism of miRNA dysregulation in CRC pathogenesis is not fully understood, but unique sets of miRNA expression profiles have been identified in a vast array of cancers, for example, ovarian, breast, and prostate. The changes in this profile during therapeutic response and disease progression provide the potential for the advancement in cancer treatments [6].

MiR-133a has been reported on several occasions to be downregulated in cancers when compared to normal adjacent tissue, and it has been implemented as a tumor suppressor targeting several oncogenes. MiR-133a targets FSCN1 in bladder cancer and CAV1 in head and neck squamous cell carcinoma functioning as a tumor suppressor [9, 10]. Many published papers involving miRNAs array data showed that miR-133a was underexpressed in CRC when compared to normal adjacent tissue, and miR-133a has also been documented to target LASP1 through the mitogen-activated protein kinase pathway (MAPK) [11]. In breast cancer, the loss of miR-133a expression has been associated with poor survival, and restoration of this expression was found to reduce cell invasion in breast cancer cell-lines and also regulate proliferation by targeting epidermal growth factor receptor [12].

## 2. Materials and Methods

### 2.1. Patients and Tissue Samples

The study included 125 patients (age 29–95 years, mean age of 71.8 years) with primary CRC diagnosed and resected at the Department of Surgery, Queen Mary Hospital, Hong Kong, between the years 2008 and 2012. For each case, samples from primary tumor and the corresponding normal colorectal mucosa were retrieved for comparison. All samples were flash-frozen in liquid nitrogen and stored at −80°C until further molecular analysis. Written consent was received for all patients who were recruited. The clinical and pathological data was obtained from the hospital records relating to age, gender, diagnosis, tumor location and size, TNM staging, local invasion, differentiation, and distant metastasis. U6 small nuclear RNA (U6) and small nucleolar RNA, C/D Box 48 (RNU48), were used as potential references genes and U6 was used for further analysis. Expression levels were “normalized” using small nuclear RNA U6 which has been used by many other studies. This study was approved by the Institutional Review Board of the hospital.

### 2.2. RNA Extraction and Quantitative RT-PCR

TaqMan microRNA assay (Applied Biosystems, Foster City, CA) was used to relatively quantify and detect the miRNA levels of miR-133a (assay ID. 002246); U6 (Assay ID. 001973) and RNU48 (Assay ID. 001006) were utilized as internal controls. Total RNA was extracted from tumor tissue and adjacent normal mucosa using mirVana miRNA isolation Kit (Ambion, Austin, TX) according to the manufacturer's instructions. The RNA concentrations were measured using Nanodrop ND-1000 spectrophotometer (Nanodrop Technologies, Wilmington, DE, USA).

This total RNA was used to reverse transcribe mRNA complementary DNA (cDNA) and miRNAS using TaqMan Microarray Assays (Applied Biosystems, Foster City, CA) from 125 patient's tissue samples. Each reverse transcriptase reaction utilized 10 ng of total RNA (5.00 *μ*L), 0.15 *μ*L dNTP (100 mM total), 1.00 *μ*L Multiscribe RT enzyme (50 U/*μ*L), 1.50 *μ*L 10X RT buffer, 0.19 *μ*L RNase Inhibitor (20 U/*μ*L), 4.16 *μ*L nuclease free water, and 3 *μ*L 5X RT primer making a total reaction volume to 15 *μ*L. Takara PCR thermal cycler DICE was programmed as follows: 30 min at 16°C, 30 min at 42°C, and 5 min at 85°C. Real-time PCR was performed using real-time PCR 7900HT system (Applied Biosystems, Foster City, CA). The reversely transcribed cDNA was diluted 1 : 20 before the qPCR reaction volume was mixed and 1.33 *μ*L was combined with 10 *μ*L 2X Universal PCR Master Mix (no AmpErase UNG), 7.67 *μ*L water, and 1.0 *μ*L 20X MicroRNA Assay. A total volume of 20 *μ*L per reaction was transferred to a 96-well MicroAmp plates (Applied Biosystems, Foster City, CA) and incubated for 10 min at 95°C, followed by 40 cycles at 95°C for 15 sec. and then 60°C for 60 sec. All samples were run in duplicate.

For miR-133a, the primers were as follows: forward, 5′-UUUGGUCCCCUUCAACCAGCUG-3′ and reverse, 5′-UAAACCAAGGUAAAAUGGUCGA-3′.

CDNA synthesis for mRNA expression was performed using Primescript RT Master Mix according to manufacturer's protocol (Takara Bio, Japan). The qRT-PCR amplification of LASP1, CAV1, FSCN1, and *β*-Actin (internal control) mRNA was performed using real-time PCR 7900HT system (Applied Biosystems, Foster City, CA) and Faststart SYBR Green PCR Master Mix (Roche Diagnostics, Mannheim, Germany) according to manufacturer's instructions. The PCR conditions were 50°C for 2 min and 95°C for 10 min, followed by 40 cycles at 95°C for 15 sec and 60 for 60 sec. The expression levels of LASP1, CAV1, and FSCN1 mRNA were normalized to *β*-ACTIN mRNA expression. The specific primers were as follows: LASP1, 5′-CTTCGCCTCAAGCAACAGAGTG-3′ (forward) and 5′-TGTCTGCCACTACGCTGAAACC-3′ (reverse); CAV1, 5-CCAAGGAGATCGACCTGGTCAA-3′ (forward) 5′-GCCGTCAAAACTGTGTGTCCCT-3′ (reverse); FSCN1, 5-GACACCAAAAAGTGTGCCTTCCG-3′ (forward) 5′-CAAACTTGCCATTGGACGCCCT-3′ (reverse) and *β*-Actin, 5′-CGAGCATCCCCCAAAGTT-3′ (forward) 5′-GCACGAAGGCTCATCATT-3′ (reverse).

### 2.3. Statistical Analysis

All relationships of the relative expression of the target miRNA were performed using Prism 5 (Graphpad, CA, USA) statistical software. *P* values are two-sided, and *P* < 0.05 were considered statistically significant. Associations between miR-133a expression and clinicopathological features were explored using Mann-Whitney *U* and Kruskal-Wallis test, as appropriate. Survival was estimated using the Kaplan-Meier method and compared using the log-rank test. Overall, metastasis-free survival and survival curves were calculated based on date of surgery till date of death or diagnosis of metastasis. Patients who had been followed up for more than 2 years or until time of death were used to calculate overall survival (OS). The expression level was considered downregulated if the fold change was lower than 0.40 or upregulated if more than 2.5, respectively. The data is shown in ΔΔ form on the figures, with higher values being indicative of lower expression, and is calculated by normalization with internal controls and normal tissue expression. Positive values represent downregulation. The relative miR-133a expression is calculated using the formula 2^−(Δct)^. Correlation between miR-133a and potential target genes was analyzed by Pearson's method; the *P* value was calculated by *F* test (Graphpad, CA, USA).

## 3. Results

### 3.1. Comparison of miR-133a Expression between Adjacent Normal Mucosa and Tumorous Tissue

**Figure 1:**
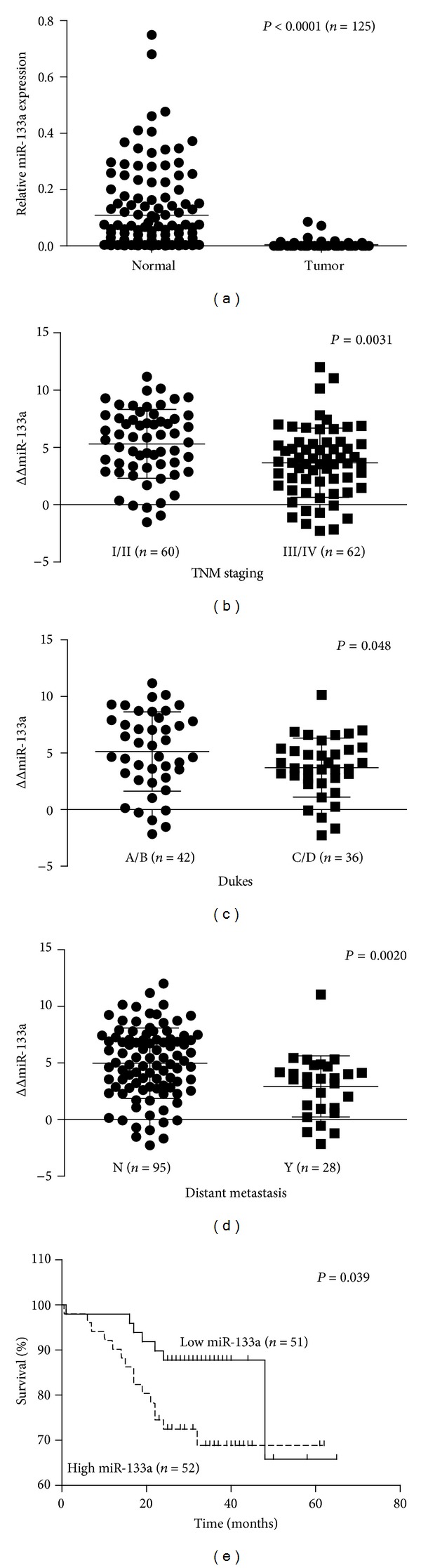
MiR-133a expression in colorectal cancer. (a) Comparison of miR-133a expression levels in 125 patients between colorectal cancer tissue and adjacent normal mucosa. The expression levels were normalized using U6 as an internal control. *P* value was calculated by Student's *t*-test. MiR-133a expression levels were downregulated in cancer tissue than in normal adjacent mucosa (*P* < 0.0001); (b) and (c) correlation between miR-133a expression and TNM and Dukes stage in patients with colorectal cancer: (b) Dukes stage (*P* = 0.048), (c) TNM stage (*P* = 0.0031). (d) Correlation between miR-133a expression and development of distant metastases in 123 patients with colorectal cancer. Difference was significant between development of distant metastases (Y) and no development of distant metastases (N): (*P* = 0.002). (e) Five-year Kaplan-Meier survival curves according to the level of miR-133a expression for colorectal. There were 103 colorectal patients that were classified into two groups according to the median miR-133a expression level as determined by qRT-PCR. MiR-133a expression was normalized to U6, an internal control. High expression group of miR-133a was correlated with significantly poorer prognosis, that is, survival rate, than in the low expression group (*P* = 0.039), *n* = number of patients. A higher ΔΔ value indicates lower expression and positive values represent downregulation 1. Relative miR-133a expression is calculated using the formula 2^−(ΔCT)^.

The expression level of miR-133a was significantly downregulated in tumor compared to adjacent nontumor mucosa (Figure 1(a), *P* < 0.0001) by a mean value of 0.04-fold difference. Among the 125 samples, 104 (83.2%) cancers displayed low miR-133a expression; of the other 21 tumors, 4 (3.2%) tumors displayed upregulated miR-133a expression and 17 (13.6%) tumors displayed no change in expression compared to adjacent nontumor mucosa, suggesting that miR-133a repression was frequently observed in CRC patients.

### 3.2. Reexpression of miR-133a in CRC Was Correlated with Advanced Staging and Development of Distant Metastases

The clinicopathological significance of miR-133a expression was determined. There was no significant correlation between miR-133a expression and other clinicopathological characteristics, such as gender, age, tumor size, histological type, tumor location, and depth of invasion (*P* < 0.05, Table 1). However, when comparing early and late TNM clinical and Dukes staging, a significant difference in miR-133a expression was found, respectively (Figures 1(b) and 1(c), *P* = 0.0031, *P* = 0.048). Advanced TNM and Dukes staging was associated with higher miR-133a expression even though its expression was mostly still, lower in tumor tissues. In addition, higher expression of miR-133a was associated with the development of distant metastases (Figure 1(d), *P* = 0.0020) in CRC patients, indicating the significance of miR-133a in tumor metastasis.

The prognosis of 101 CRC patients whose progress was followed after surgical resection was also analyzed. Based on their median value of miR-133a expression level, the outcomes of the patients were divided into 2 groups, high and low miR-133a expression, and were shown to have a significant difference using Kaplan-Meier survival analysis and Gehan-Breslow Wilcoxon test (Figure 1(e), *P* = 0.039). Patients with a higher expression of miR-133a had a significantly lower rate of overall survival than the patients with low miR-133a expression.

### 3.3. Downregulated miR-133a Expression Was Correlated with Several Potential Target Genes Related to Metastasis

**Figure 2:**
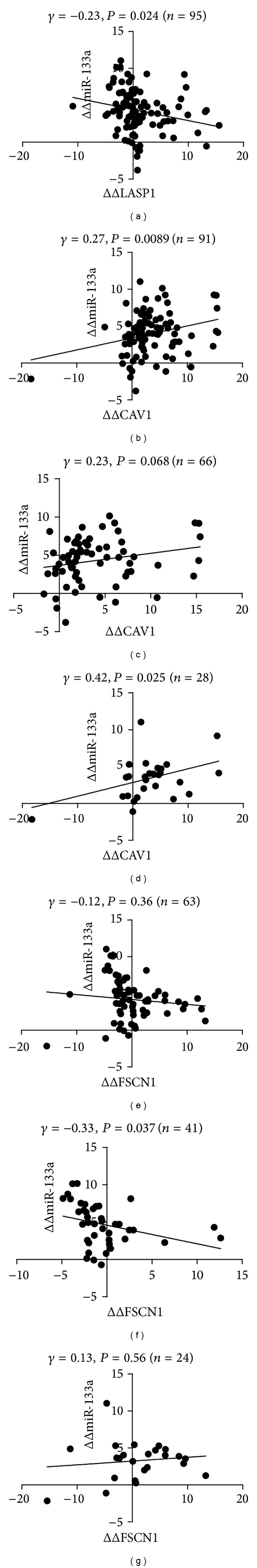
Correlation of miR133a and its potential target genes in colorectal cancer. (a) There was a significant negative correlation shown between miR-133a and LASP1 mRNA expression: *P* = 0.024, *γ* = −0.23, (*n* = 95). (b)–(d): (b) An overall significant correlation was found between miR-133a and CAV1 mRNA expression level: *P* = 0.0089, *γ* = 0.27, (*n* = 91), (c) nonmetastatic: *P* = 0.068, *γ* = 0.23, (*n* = 66), and (d) metastatic, a stronger positive correlation was determined between patients whom develop distant metastases: *P* = 0.025, *γ* = 0.42, (*n* = 28). (e)–(g): (e) overall: *P* = 0.037, *γ* = −0.33, (*n* = 63), (f) nonmetastatic, a significant negative correlation was shown between miR-133a and FSCN1 mRNA expression levels that have not yet developed metastases: *P* = 0.037, *γ* = −0.33, (*n* = 41), (g) metastatic: *P* = 0.56, *γ* = 0.13, (*n* = 24), *n* = number of patients. Higher ΔΔ values indicate lower expression; positive values represent downregulation.

Overall, in 95 patients whose LASP1 and miR-133a expression were analyzed, a negative correlation (Figure 2(a), *γ* = −0.23, *P* = 0.024) was found, suggesting that LASP1 was indeed negatively regulated by miR-133a in CRC. Interestingly, there was a positive correlation between CAV1 and miR-133a expression among CRC patients (Figure 2(b), *γ* = 0.27, *P* = 0.0089). Additionally, there was a stronger positive correlation between miR-133a and CAV1 expression in patients who developed distant metastases (Figure 2(c), *γ* = 0.42, *P* = 0.025). In patients who did not develop metastases, there was a significant negative correlation between FSCN1 and miR-133a expression (Figure 2(f), *γ* = −0.33, *P* = 0.037), but no significant association was observed in patients with metastatic disease. These results indicated the complex mechanism of target genes regulated by miR-133a, in which the miR-133a regulatory effect was opposite to that reported in other studies (CAV1), or even within CRC of different metastatic potential (CAV1 and FSCN1).

## 4. Discussion

Even though miR-133a expression was, for the most parts, downregulated in tumor tissue, the higher expression among patients correlated with poor prognosis and adverse clinical characteristics. The correlation of miR-133a expression with the development of distant metastases as well as advanced TNM and Dukes staging indicates that miR-133a expression is associated with metastasis and hence tumour progression of CRC.

Many functional studies have been conducted on miR-133a and its experimentally proven oncogene targets, suggesting its role as a tumor suppressor in various cancers [9, 13, 14]. However, limited studies have been conducted on correlation between clinical data and miR-133a expression in large cohorts of patients, indicating a lack of clinicopathological knowledge regarding miR-133a in CRC and limited attempts to identify its direct targets in clinical samples. In molecular studies, miR-133a has been shown to directly regulate several metastasis-related oncogenes, including FSCN1 in bladder cancer [9], LASP1 in CRC [11], and CAV1 in head and neck squamous cell carcinoma [15]. There are conflicting reports regarding the role of miR-133a in cancer progression as gain-of-function studies have been shown to inhibit metastasis and tumorigenesis using molecular studies and animal models in CRC [6].

This study found that CAV1 was not directly targeted by miR-133a in CRCs as there was an overall positive correlation between their miR-133a and CAV1 expression (Figure 2(b), *γ* = 0.27, *P* = 0.0089), and even stronger correlation in patients who developed distant metastases (Figure 2(d), *γ* = 0.42, *P* = 0.025). This change in correlation between CAV1 and miR-133a expression implies dysregulation of either one or both of these genes, providing additional evidence to previous papers regarding the reexpression of CAV1 increasing metastatic and migratory potential. The evidence for the dysregulation of miR-133a during metastasis is similar to CAV1 in that it is reactivated during metastasis since its expression changes during tumor progression [16–18]. CAV1 has been characterized to possess a dual-function in cancer progression as a tumor suppressor and oncogene, and its reexpression has been implied to be necessary for metastasis in certain types of cancer to promote cell migration and invasion, for example, breast, lung, and prostate [19–21]. These reactivation and upregulation have been indicative of more aggressive and chemoresistant phenotypes in some cancers with poor prognosis [22, 23]. The contrasting functions for CAV1 were partially explained by Williams and Lisanti who observed CAV1 to possess several peptides regions with different roles [16]. Previously, CAV1 has been reported to be downregulated in CRC and possessed “differential biphasic expression” which supports the findings that CAV1 is positively correlated with miR-133a in CRC patients, particularly in those who developed distant metastases, since advanced tumor progression and metastasis were associated with higher miR-133a expression as demonstrated in this study [17, 18, 24].

MiR-133a expression was found to be inversely correlated with LASP1 expression (Figure 2(a), *γ* = −0.23, *P* = 0.024) corroborating the previous study by Wang et al. regarding LASP1 being a direct target of miR-133a in CRC in their functional study involving an orthotropic model [25]. Interestingly, LASP1 gene has previously been reported to be overexpressed in CRC possessing a functional role in tumor metastasis and progression, and its overexpression in metastatic breast and ovarian cancer further supported this [11, 26]. On the contrary, FSCN1 was only found to be negatively correlated in patients who have not yet developed metastases (Figure 2(f), *γ* = −0.33, *P* = 0.037), suggesting that FSCN1 is dysregulated by miR-133a and its suppression impaired metastasis. FSCN1 has been proven to be a metastatic gene and target of miR-133a in esophageal squamous cell carcinoma and bladder cancer [9, 10]. The functional role documented by many papers indicates that it is generally involved in the migration and invasion and, therefore, involved in the seeding of circulating tumor cells to metastatic sites, hence explaining its suppression by miR-133a in nonmetastatic patients [27, 28]. In CRC, miR-451 has also been proven to mediate FSCN1 expression by inhibiting 5′ AMP-activated protein kinase (AMPK) when overexpressed, which in turn activates the mammalian target of rapamycin (mTOR) that is capable of regulating FSCN1 [29]. This indicates that at least two microRNAs regulate FSCN1 expression in CRC, providing an explanation why miR-133a expression correlates with FSCN1 only in nonmetastatic CRC patients.

Higher expression of miR-133a has also been linked to increased metastasis in research conducted by Nohata et al., who found miR-133a to be a promoter of genes related to increased brain metastasis in CRC [30]. Based on this previous paper, the miR-133a expression not only increases in the primary tumor but also in the metastases, in which miR-133a expression is higher than the primary.

Higher expression of MiR-133b, a homologue of miR-133a which shares the same transcription unit, was shown to be associated with poorer survival when compared to low expression in 106 bladder cancer patients (*P* < 0.001) indicating the potential for this group of miRNAs to function as oncomirs [31]. MiR-133a and miR-133b have been shown to both target FSCN1 in esophageal squamous cell carcinoma that could potentially be mirrored in CRC as they share several other potential target genes due to their high similarity in sequence, that is, only differing by one nucleotide [10, 30].

In this study, higher expression of miR-133a significantly correlated with the shorter OS in a 5-year survival analysis of 102 CRC patients, indicating the potential of miR-133a as a prognostic marker in CRC therapy. High expression seemed to be a significant predictor for poor prognosis and development of metastasis. MiR-133a was found to be upregulated in 5-fluorouracil chemoresistant cells and potentially possess a higher proportion of cells with cancer stem cell phenotype and therefore possess a higher metastatic potential based on Dallas et al. [31, 32]. Interestingly, the expression of miR-133a was also found to be upregulated in CRC patient's plasma samples compared to neoplasm-free controls, which also contradicts many tissue-based studies, indicating that miR-133a is not simply a tumor suppressor as many papers reported [33].

In conclusion, the expression of miR-133a is downregulated during CRC carcinogenesis, but its high expression in tumor tissues is correlated with advanced Dukes and TNM stage, development of distant metastasis, and poor prognosis. Therefore, miR-133a possesses the potential to function as a clinical marker to identify patients with aggressive CRC whose survival rate is likely to be low and who are prone to develop distant metastases. Also, miR-133a expression was negatively correlated with FSCN1 expression in patients who did not develop distant metastases, but positively correlated with CAV1 expression in patients who did develop distant metastases. This indicates that the role of miR-133a is much more complicated than simply a tumor suppressor. Further research is required to understand the role of miR-133a in tumor progression and its complicated interactions with oncogenes and tumor suppressors.

## Figures and Tables

**Table 1:** Association of clinicopathological factors in colorectal cancer patients and expression of mir-133a in tumor tissue. A higher ΔΔ value indicates lower expression; positive values represent downregulation.

Variables	Patients, *n*	MiR-133a expression	*P* value^†^
	(*n* = 125)^a^	Mean ΔΔ value ± SD	
Age			0.938
≤65	36	4.440 ± 0.4927	
>65	89	4.488 ± 0.3301	
Gender			0.977
Male	73	4.517 ± 0.3569	
Female	51	4.501 ± 0.4332	
Dukes			0.0481∗
A, B	42	5.139 ± 0.5392	
C, D	36	3.716 ± 0.4348	
Tumor size			0.2643
<5 cm	68	4.739 ± 0.3548	
≥5 cm	55	4.124 ± 0.4228	
Histological type			0.3664
Well, moderate	78	4.681 ± 0.3499	
Poor, mucinous	5	3.395 ± 1.172	
Depth of invasion			0.2158
T1, T2	16	5.400 ± 0.7513	
T3, T4	100	4.352 ± 0.3146	
TNM stage			0.0031∗
I, II	60	5.316 ± 0.3887	
III, IV	62	3.667 ± 0.3835	
Location			0.9658
Colon	90	4.527 ± 0.3259	
Rectum	32	4.554 ± 0.5298	
Lymph node metastasis			0.0595
Absent	68	4.926 ± 0.3629	
Present	55	3.893 ± 0.4034	
Distant metastasis			0.0020∗
Absent	95	4.983 ± 0.3188	
Present	28	2.932 ± 0.5104	

^a^The total number of cases may be less than 125 as some information was unavailable.

^†^ *P* values are based on Student's *t*-test.

SD: standard deviation; *P < 0.05.
